# An international registry for neurodegeneration with brain iron accumulation

**DOI:** 10.1186/1750-1172-7-66

**Published:** 2012-09-17

**Authors:** Bernadette Kalman, Ronald Lautenschlaeger, Florian Kohlmayer, Boriana Büchner, Thomas Kmiec, Thomas Klopstock, Klaus A Kuhn

**Affiliations:** 1 Friedrich-Baur-Institut, Dept. of Neurology, Ludwig-Maximilians-University Munich, Ziemssenstraße 1a, Munich, 80336, Germany; 2 Institut für Medizinische Statistik und Epidemiologie, Klinikum Rechts der Isar der Technischen Universitaet, Ismaninger Str. 22, Munich, 81675, Germany; 3 Instytut Pomnik Centrum Zdrowia Dziecka, Klinika Neurologii I Epileptologii, Aleja Dzieci Polskich 20, Warszawa, 04-730, Poland

**Keywords:** Rare diseases, Neurodegeneration, Iron, Registry, Consortium, NBIA, TIRCON

## Abstract

We report the development of an international registry for Neurodegeneration with Brain Iron Accumulation (NBIA), in the context of TIRCON (Treat Iron-Related Childhood-Onset Neurodegeneration), an EU-FP7 – funded project. This registry aims to combine scattered resources, integrate clinical and scientific knowledge, and generate a rich source for future research studies. This paper describes the content, architecture and future utility of the registry with the intent to capture as many NBIA patients as possible and to offer comprehensive information to the international scientific community.

## 

Rare mendelian disorders are being characterized with increasing success owing to the rapid development of tools for defining phenotypic and genetic determinants. The elucidation of disease pathogenesis may simultaneously trigger the identification of pathway-specific treatment targets, but the development of disease-modifying medications is often hampered by the fragmentation of efforts and the low number of available patients. The integration of scattered resources is, therefore, crucial for the success of future scientific accomplishments. Here we report the inception of a central data repository for a group of rare disorders collectively called Neurodegeneration with Brain Iron Accumulation (NBIA).

NBIA has an estimated prevalence of 1–3 / 10^6^ and consists of several genetically-defined entities that clinically often overlap but also have unique features (Table 1). The neuropathological hallmarks include iron accumulation associated with neuronal loss and gliosis, which appear as hypointense lesions (iron) with or without hyperintense patches (gliosis) on T2-weighted MRI in most NBIA forms. The “eye of the tiger” sign (a hypointense area with a hyperintense center) in the globus pallidus is seen in Pantothenate Kinase-Associated Neurodegeneration (PKAN) ( [1]). Iron in the brain can be assessed by using dual spin-echo and multiple gradient-echo MRI sequences, which allow monitoring an endophenotype of the disease. Biological heterogeneity of NBIA is reflected by the differential appearance of various neuronal inclusions in the genetically defined sub-entities. However, shared functions of the affected genes are also recognized in the maintenance of membrane phospholipids and the regulation of mitochondrial energy metabolism, explaining the degenerative features of neuropathology ( [2]). Even though the excess of iron appears pathogenic, molecular causes and consequences of its accumulation remain unknown. An iron-chelating agent, deferiprone, approved for systemic iron-overload disorders, is currently being tested in the largest subgroup of NBIA, PKAN (Table 1). This deferiprone trial with the associated biobank and patient registry represent core work packages in TIRCON (Treat Iron-Related Childhood-Onset Neurodegeneration), an international consortium supported by the European CommissionÂ´s Seventh Framework Programme. 

**Table 1:** NBIA disorders

**Disease**	**Gene**	**Inheritance**	**Clinical presentation**	**MRI**
**Classic form:** Early onset – rapid progression				
Pantothenate kinase-associated neurodegeneration (PKAN)	*PANK2*	autosomal recessive	Gait abnormality, dystonia, dysarthria, corticospinal involvement, pigmentary retinopathy	“Eye-of-the-tiger” sign in the GP
Mitochondrial membrane protein-associated neurodegeneration (MPAN)	*C19orf12*	autosomal recessive	Speech and gait difficulties, progressive spastic paraparesis, dystonia, optic atrophy, axonal motor neuropathy, cognitive decline	Increased iron in GP and SN; “Eye of the tiger” sign very rare
Fatty-acid hydroxylase-associated neurodegeneration (FAHN)	*FA2H*	autosomal recessive	Onset with focal dystonia and gait impairment, ataxia, dysarthria, spastic quadriparesis, nystagmus, visual loss, no or mild cognitive impairment and seizures in late stages; overlaps with some HSP syndromes and phenotypes of leukodystrophies	Abnormal iron in GP and SN, optic, cerebellar and brainstem atrophy, confluent subcortical and periventricular hyperintensity, thinning of the CC
PLA2G6-associated neurodegeneration (PLAN) –Infantile neuroaxonal dystrophy (INAD)	*PLA2G*	autosomal recessive	Severe psychomotor regression, hypotonia, peripheral motor neuropathy, hyperreflexia, tetraparesis, ataxia, gait abnormalities	Cerebellar atrophy, abnormal iron in GP and other nuclei
**Atypical form:** Later onset – slower progression				
Atypical pantothenate kinase-associated neurodegeneration (PKAN)	*PANK2*	autosomal recessive	Speech abnormalities, depression, impulsivity, aggression, emotional instability	Eye-of-the-tiger sign or variants of it in the GP or other signs of increased iron and gliosis in the GP
Atypical mitochondrial membrane protein-associated neurodegeneration (MPAN)	*C19orf12*	autosomal recessive	Psychiatric features, parkinsonism, dystonia-parkinsonism, motor axonal neuropathy, mild gait difficulty, optic atrophy, cognitive decline	Increased iron in GP and SN
Neuroferritinopathy	*FTL*	autosomal dominant	HuntingtonÂ´s disease-like presentation: adult-onset chorea or dystonia, orofacial action dystonia, cognitive decline	Abnormal iron and later cystic changes in the basal ganglia
Aceruloplasminemia	*CP*	autosomal recessive	Diabetes mellitus, retinal degeneration, blepharospasm, facial and neck dystonia, chorea, tremor, dysarthria, ataxia – typically adult onset	Pathological iron in both the brain and viscera; in the brain: GP, striatum, thalami, dentate nuclei
				In the viscera: liver
PLA2G6-associated neurodegeneration (PLAN) –neuroaxonal dystrophy (NAD)	*PLA2G6*	autosomal recessive	Progressive dystonia, dysarthria, language delay, corticospinal signs, psychiatric features and social difficulties or similar to but later onset version of INAD	Cerebellar and optic nerve atrophy
				Iron may or may not be increased
PLA2G6-associated neurodegeneration (PLAN) – dystonia-parkinsonism	*PLA2G6*	autosomal recessive	Parkinsonism, dystonia-parkinsonism	Iron may be increased in SN an striatum
**Other NBIA form**				
Kufor-Rakeb syndrome	*ATP13A2*	autosomal recessive	Juvenile onset parkinsonism, corticospinal signs, supranuclear gate palsy and cognitive decline, facial-faucial-finger myoclonus, visual hallucinations, oculogyric crisis	Generalized brain atrophy, increased iron in the caudate and lenticular nuclei, but increased iron is not always present
Woodhouse-Sakati Syndrome	*C2orf37*	autosomal recessive	Hypogonadism, diabetes, alopecia, hearing loss, mental retardation, progressive generalized and focal dystonia, dysarthria, cognitive decline	Increased iron in GP, SN and other nuclei; white matter abnormalities
Static Encephalopathy with Neurodegeneration in Adulthood (SENDA)	*In progress*	autosomal recessive	Childhood onset cognitive impairment without progression; in adult age sudden onset progressive dystonia-parkinsonism and corticospinal signs.	Increased iron in the GP and SN; on T1W MRI, hyperintensity with central band of hypointensity in the SN; cerebral and cerebellar atrophy

Legend to Table.

The table summarizes the currently known entities collected under the umbrella of NBIA. Transitional phenotypes are increasingly observed within the spectrum between the extremes of the early and late onset forms due to the routine use of molecular genetics in clinical practice.

Increased iron in the brain is reflected by hypointensity on T2-weighted MRI scans, and is typically detected in nuclei where some iron is normally present: GP, SN, putamen, caudate, dentate nuclei and red nuclei. The “eye of the tiger” sign refers to an ovoid area of hypointensity with a hyperintense center corresponding to increased iron and gliosis, respectively, within the GP. The histological substrates of iron accumulation include the astrocytes, microglia / macrophages, neurons, neuropil and the perivascular space. In the same regions, axonal spheroids, neuronal loss, gliosis and demyelination may also be present. Ceroid lipofuscin and neuromelanin may accumulate intracellularly and extracellularly. The numbers of histopathological studies on genetically defined subentities are limited, but so far have revealed a differential occurrence of various neuronal inclusions.

GP: Globus pallidus.

SN: Substantia nigra.

CC: Corpus callosum.

T1W MRI: T1-weighted magnetic resonance imaging.

T2W MRI: T2-weighted magnetic resonance imaging.

NA: not available.

As a central data repository of TIRCON, the NBIA registry ( http://www.tirconregistry.org) manages links to the deferiprone trial database, the NBIA image archive and biobank. The collected information includes demographics, family and patient history, clinical signs, neurological exam, laboratory results, brain iron assessment and volumetry by imaging, treatments and trials. The disease course is captured by standardized scales measuring dystonia severity, quality of life and sleep, functional independence, cognition, motor functioning and activity of daily living. The annually entered information enables a longitudinal reconstruction of the disease course, while genomic, transcriptomic, proteomic and metabolomic data from research on the biobank specimens contribute additional dimensions to the registry.

The technical development of the registry involved significant preparatory works. During an initial phase of six months, existing local databases and their datasets were analysed in order to specify data elements which are harmonized and most useful for the international NBIA registry. For quick and easy data entry by participating clinicians, web-based forms have been created. Management of directly identifying data are restricted to the respective sites, and only patient codes (pseudonyms) will be entered into the registry database. Together with these patient pseudonyms, codes identifying biospecimens, images, and data in the deferiprone trial database are kept in the registry and safeguarded by a sophisticated security and privacy architecture.

The registry’s security and privacy concept builds upon the security architecture of the German mitoNET repository ( [3]), which has been successfully running for more than three years. Access rights are strictly role-based, and pseudonyms are kept spatially and organizationally separate from the registry database storing medical/research data. Double coding is used for all pseudonyms, which are additionally secured by encryption. In terms of the US Health Insurance Portability and Accountability Act, protected health information compliant with the “limited data set” definition is stored.

For the use of data and biosamples, agreements have been specified and approved by local ethics committees and data protection authorities. Future research proposals will be reviewed by the TIRCON’s Scientific Steering Committee (SSC). If the proposal is approved, pseudonymous or anonymous data will be released to the future investigator, depending on the proposal and the vote of the SSC. As an additional safeguard, re-identification attempts are prohibited by the data use agreements.

In summary, this registry longitudinally collects multi-dimensional information on NBIA disorders to provide a rich resource for future natural history studies, evaluations of clinical and paraclinical surrogates or potential predictors of the disease, and correlation analyses of clinical subentities with pathogenic mutations, alleles of disease modifying genes, results of “-omics” and environmental factors. It will support the design of placebo-controlled, randomized, double-blind clinical trials, and allow probing which clinical, imaging or laboratory features of the disease may be modified by a treatment modality. The NBIA registry represents a paradigm fostering integration of international resources for the enhancement of scientific synergism and future research activities in a group of rare diseases.

## Competing interest

Dr. Bernadette Kalman – Received travel support and honoraria for giving lectures on MS from Biogen-IDEC and Novartis; Serves as an Associate Editor for BMC Neurology; Received research grants from the NMSS and EMD Serono in the past; Dr. Klaus Kuhn - serves/has served on the Editorial Boards of *Methods of Information in Medicine*, the *International Journal of Medical Informatics*, *it – Information Technology*, and *Informatics*. He serves on the Scientific Advisory Board of OFFIS, Oldenburg, and he has served for the Austrian Accreditation Council. He receives funding from BMBF grants 01 GM 1113C, 01 KN 1104, 01 EX 1020 E, Z76010067200, 80005012, and EU grants 284209, 277984; Ronald Lautenschlaeger – Receives personal compensation from TMF e.V.: Umbrella Organization for Networked Medical Research in Germany; Dr. Thomas Klopstock - Has received research support from government entities (Deutsche Forschungsgemeinschaft, Bundesministerium für Bildung und Forschung, European Commission 7th Framework Programme) and from commercial entities (Santhera Pharmaceuticals Ltd, Actelion Pharmaceuticals Ltd, H. Lundbeck A/S); Has been serving on scientific advisory boards for commercial entities (Santhera Pharmaceuticals Ltd; Actelion Pharmaceuticals Ltd) and for non-profit entities (Center for Rare Diseases Bonn, Germany; Hoffnungsbaum e.V., Germany). He has received speaker honoraria and travel costs from commercial entities (Dr. Willmar Schwabe GmbH & Co. KG; Eisai Japan, Santhera Pharmaceuticals Ltd, Actelion Pharmaceuticals Ltd). He has been doing consultancies for the Gerson Lehrman Group, USA, and has been serving as a Section Editor for BMC Medical Genetics from 2011. Other co-authors (BB, FK, TKm) declare that they have no competing interests.

## Authors’ contributions

Bernadette Kalman: Drafted main part and created the final version of the manuscript, designed the clinical and scientific content of the registry.

Ronald Lautenschlaeger: Implemented the content and participated in the design of the IT – architecture and data security concepts of the registry, reviewed and revised the manuscript.

Florian Kohlmayer: Participated in the design of the IT - architecture and the data security concept of the registry, reviewed and revised the manuscript.

Boriana Buechner: Reviewed and revised the manuscript.

Tomasz Kmiec: Contributed to the content of the registry, reviewed and revised the manuscript.

Thomas Klopstock: Principal investigator for TIRCON. Contributed to the content design of the registry, reviewed and revised the manuscript.

Klaus Kuhn: Designed the IT - architecture and data security concept of the registry, drafted part of the manuscript, reviewed and revised the final manuscript.
